# Reviewers acknowledgement

**DOI:** 10.1002/1878-0261.12625

**Published:** 2020-01-06

The Editors of *Molecular Oncology* would like to thank all referees who have given their time and expertise to review articles submitted for publication in Volume 13. *Molecular Oncology* heavily relies on reviewers' careful assessment, constructive criticism and timely response to ensure both quality and fast turnaround times.

The names of these reviewers are listed below; we apologize if we have inadvertently omitted anyone.

Alejandro Pablo Adam

Alejandro Athie

Sam Bunting

Sylvie Deborde

Pedro M Enriquez‐Navas

Ceres Fernández Rozadilla

Emily Keung

Vakul Mohanty

Mark Adams

Neerag Agarwal

Andreas Agathangeliidis

Massimo Aglietta

Massimiliano Agostini

Rosemary Akhurst

Yoshimitsu Akiyama

Donatella Aldinucci

Raquel Almeida

Maria Carmen Alpoim

Ivano Amelio

Christopher I Amos

Chris Amos

Kenneth Anderson

Simone Anfossi

Lindsay Angus

Toni Antalis

Emmanuel Stylianos Antonarakis

Sandra Appiah

Yannick Audet‐Delage

PP Aung

Timucin Avsar

Ninel Azoitei

J Bae

Marta Baiocchi

Justin Balko

Sulagna Banerjee

Aria Baniahmad

Marco Barchi

Alberto Bardelli

Marin Barisic

Dalia Barsyte

Jiri Bartek

Michael Barton

Cristina Battaglia

Recep Bayraktar

James Bayrer

Yaacov Ben‐David

Virginie Bernard

Rene Bernards

Alexander Berndt

Anton JM Berns

E Penni Black

Giovanni Blandino

Cedric Blanpain

Yuna Blum

Mattia Boeri

Victor M Bolanos‐Garcia

Joanna Boncela

Laura Bongiovanni

Shirin Bonni

Carl AK Borrebaeck

Francisco Borrego

Guillaume Bossis

Roland P Bourette

Michael Boutros

Cornelia Braicu

Douglas Brash

Jonathan Brody

Christian Bronner

LAA Brosens

Kristin Brown

Maike Buchner

Martin Bushell

Zhixiong Cai

Leyi Cai

Hao Cai

George Calin

Marco A Calzado

Ettore Domenico Capoluongo

Vera Cappelletti

Andres Cardona

Aura Carreira

Jason Carroll

J Ignacio Casal

Sergi Castellvi‐Bel

Sonia Castillo‐Lluva

Julio Celis

Ariana Centa

Andres Cervantes

Kishore Challagundla Babu

Suhwan Chang

Guoqiang Chang

Samit Chatterjee

Yun Che

Ting Chen

Hongwei Chen

Dung‐Tsa Chen

Guan Chen

Zhicong Chen

Linxi Chen

Wannan Chen

Yi‐Rong Chen

Jason Chia‐Hsien Cheng

Samuel H Cheshier

Keeming Chia

Frederic Chibon

HA Chiu

Chih‐Hung Chou

Markus Christmann

Wm Chu

Valentina Cianfanelli

Roberta Ciarapica

Berta Cillero

Yari Ciribilli

Maria Ciriolo Rosa

Peter Clark

Robert Clarke

Florian Clatot

David Cobrinik

Maria Colombino

Robert Coppes Paul

Rosalia Cordo Russo

Bruno Costa Da Silva

Joseph Costello

Francesco Crea

Emanuele Crespan

William Cress Douglas

Ana B Crujeiras

Jiefeng Cui

Nicola Curtin

Zhi Dai

Ting Dai

Giovanna Damia

Vincenzo D'angiolella

Daniel Danila

Dalila Darmoul

Zbigniew Darzynkiewicz

Elai Davicioni

Leonor David

James Davies

Kenneth A Dawson

Olivier De Wever

Meike De Wit

Daniela De Zio

Giannino Del Sal

Romain Désert

Aaron Diaz

Frank Diehl

Georgios N Dimitrakopoulos

Zonghui Ding

Luc Dirix

Sophia Doll

Susan J Done

Hu Dong

Mihnea Dragomir

Mihnea P Dragomir

JJ Driscoll

Peter Droege

Chong Du

Yuxin Du

Ligin Du

AC Dudley

M Dyer

Jerome Eeckhoute

Robert Eferl

Gerda Egger

Alexander Eggermont

Shogo Ehata

Sara Elgamal

Asmaa El‐Kenawi

N Erin

Maria Escalante‐Perez

Irene Esposito

Stephen P Ethier

Matthias Eyrich

Weiyi Fang

Grazia Fazio

Yu‐Mei Feng

Sandra Fernandez

Jose Fernandez Piqueras

Oscar Fernandez‐Capetillo

Manuela Ferracin

Andreas Fielding

Giuseppe Filomeni

Gian Maria Fimia

James M Flanagan

Thomas Fleischer

Tullio Florio

Gilles Flouriot

Emmanouil Fokas

John G Foley

Anthony Ford

Pietro Formisano

Albert Fornace Joseph

Daniel Frigo

Satoshi Fujii

Brian Gabrielli

Udo S Gaipl

Jin Gao

Julie Gavard

Michael H Gerber

Patrizio Giacomini

Olivier Gires

Michal Goldberg

Lauren Gollahon

Marta Gómez De Cedrón

Yusuke Goto

Tyson Graber

G Grignani

Sergei Grivennikov

Irina Gromova

Julia Gross

Yunyan Gu

Thierry Guillaume

Fabienne Guillaumond

Wei Guo

X Guo

Z Guo

Sanjay Gupta

Tony Gutschner

David Guttery

Thanos D Halezonetis

C Hallas

Petra Hamerlik

Gianna Hammer

George Hammons

Junwei Han

Jonathan Harris

Wolfgang Hartmann

Kieran Harvey

Masamichi Hayashi

Ingrid Hedenfalk

Hakan Hedman

Ellen Heitzer

Luisa Helguero

Åslaug Helland

A Helland

Raymond Henderson

Fernando Gomez Herreros

Dov Hershkovitz

Jochen Hess

Jörg D Hoheisel

Jian Hou

Tsung‐I Hsu

Jing Hu

Gang Huang

Jian Huang

Pintong Huang

Tao Huang

Petra Huehnchen

Kam M Hui

Shingo Ichimiya

Natalia Ignatenko

H Inoue

Osamu Ishibashi

Yuichi Ishikawa

Ashok Jain

Anna Janaszewska

Melanie Janning

Siegfried Janz

Seung Min Jeong

Bai Ji

Shuheng Jiang

Li Jiang

Connie R Jimenez

Sebastien Jo

Andreas Joerger

Faye M Johnson

Kyle Jones

VC Jordan

Nicolai Juul Birkbak

Deepak Kanojia

Mohammed Kashani‐Sabet

M Katoh

Ahmed Katsha

Julhash U Kazi

Kun Ke

Ahmad Khalil

Ja‐Eun Kim

Dongbum Kim

Michael Kirschfink

Christiane Klec

Carolyn M Klinge

Thomas Klonisch

Sebastian Kobold

Dimas Konstantinos

Harley I Kornblum

Paivi J Koskinen

Frank AE Kruyt

Dieter Kube

Maria Kukuruzinksa

Prashant Kumar

Akira Kurozumi

Ulrike Kusebauch

Joshua Labaer

Ashish Lal

Hind Lal

John M Lambert

Julie Lang

Matthias Lauth

Pedro A Lazo

Terence KW Lee

Yu L Lei

Keren Levanon

H Li

Chang Li

Xinying Li

Zongfang Li

Jun Li

Zhuorong Li

Peifeng Li

Yuan Li

Pengchao Li

Qing Li

Shengwen Calvin Li

Zhiyong Liang

Evi Lianidou

Wen‐Ting Liao

Julien Licchesi

Matteo Ligorio

Zou Lin

Zhenhua Lin

Xinjian Lin

Zhihua Liu

Zhonghua Liu

Z Liu

Juan Liu

Stanley K Liu

Jinping Liu

Benjamin Lok

Weiwen Long

Chris Lord

Jingli Lu

Yuanyuan Lu

Yong‐Jie Lu

Yibao Ma

Jean‐Pascal Machiels

Salvador Macip

Libor Macurek

Roberto Madeddu

Ana Magalhães

Marcos Malumbres

Dario Marchetti

Sergio Marchini

Alberto Marini

Maritza Jaramillo

Dana Marshall

Margarita Martin Andorra

Maria De La Luz Martínez‐Chantar

Flaviana Marzano

Andrew Massey

Rebecca Mather Louise

Ander Matheu

Giuseppe Matullo

Nirmala Mavila

Apolinar Maya‐Mendoza

Martina Mcdermott

James D Mckay

Jan Paul Medema

Franziska Meier‐Stiegen

Gerry Melino

Xiaodan Meng

Paula Michea

Patrick Micke

Gerard Minuesa

Kohei Miyazono

Keiko Mizuno

Raj K Mo

Mauro Modesti

F Mollinedo

Chrit Moonen

Jose Moreira

Carlos Moreno

Milana Morgenstern

Stefan Muller

Muhammed Murtaza

Paola Muti

Maria Mycielska

Tomoe Y Nakamura

Muhammad Nawaz

Barry D Nelkin

Raphael Nemenoff

Tony TC Ng

Jie Ni

Maria Victoria Nicklison‐Chirou

Sara Nicolai

Kari Nielsen

Mef Nilbert

Yosui Nojima

Nicola Normanno

Håkan Nystrøm

Ina Oehme

Motoi Ohba

Masahisa Ohtsuka

Moshe Oren

Geoffrey R Oxnard

Navjot Pabla

Michele Pagano

Richard Palmqvist

Georgios Pampalakis

Zui Pan

Emanuele Panatta

Klaus Pantel

R Panzer‐Gruemayer

Attila Patocs

Abraham Pedroza‐Torres

Oula Penate Medina

Han Peng

Yong Peng

Rosa Peracaula

Sven Perner

Angelo Peschiaroli

Joanna J Phillips

Mauro Piacentini

Martin Pichler

Marco Pieraccioli

Jean ‐Yves Pierga

Mariaelena Pierobon

Christian Pilarsky

Karen E Pollok

Evon Poon

Tomasz Powrozek

Perla Pucci

M Pujana

Peter Natesan Pushparaj

Lishuang Qi

Hu Quidong

Anna Maria Rachiglio

Krishnaraj Rajalingam

Vijay Ramaswamy

Ana Ramírez De Molina

Benedikt Rauscher

Matthias Reeh

Sergio Rey

Sara Rhost Johanna

Masaki Ri

Jordi Ribera

Sabine Riethdorf

Ulrik Ringborg

Helen Rizos

Dd Roberts

Stefan Roberts

Simon P Robinson

Sandra Rodriguez

Fausto Rodriguez

Nieves Rodriguez‐Henche

Peter Rogan

Lars Ronnstrand

Anatoly B Rosenfeld

Rossella Rota

Barak Rotblat

Dominic Rothwell Graham

Alessandro Rufini

Robin MH Rumney

Antonio Russo

Yury Saenko

Pipsa Saharinen

Morgan Sammons

Maurizio Scaltriti

Oliver Schilling

Rainer Schindl

Thomas Schlange

Fernando C Schmitt

Ed Schuuring

Albrecht Schwab

Michal R Schweiger

Andreas Scorilas

Clare Scott

Christian Seiser

Naohiko Seki

Yoshitaka Sekido

Ge Shan

Shyam Sharan

Jason Sheltzer

Jing‐Wen Shih

Andres Shih

Masashi Shiiba

Kosuke Shimizu

T Shimo

Ewa Sikora

Rajeeva Singh

Neeraj Sinha

Jens Siveke

Artem Smirnov

Bozena Smolkova

Elizabeth Smyth Smyth

Anette Sommer

Jaewhan Song

Chunhua Song

Libing Song

Laura Soucek

Sofia Sousa

Vaidotas Stankevicius

Gt Stathopoulos

J Stebbing

Dagmar Stoiber

Carmine Stolfi

Flavie Strappazzon

Klaus Strebhardt

Makoto Sugaya

Naotoshi Sugimoto

XY Sun

Yinghao Sun

Donglin Sun

Tao Sun

Jun Sun

Silvia Surinova

Santos A Susin

Masataka Suzuki

Anita Sveen

Claudio Talora

Aik‐Choon Tan

Fuchou Tang

Hailin Tang

Nilgun Tasdemir

Kristin Tasken

Stefan Taubert

Ronald B Taylor

Ronald P Taylor

Joyce Taylor‐Papadimitriou

Irmgard Tegeder

Peter Ten Dijke

Ugo Testa

Charles Theillet

Bernard Thienpont

Alain Thierry

Sufi Thomas Mary

Michelle L Thomas

John Thompson

Martin Thurnher

David J Timson

Trent E Tipple

Ka‐Fai To

Luis Toledo

RAEM Tollenaar

Shuta Tomida

Hiroyuki Tomita

Tiziana Triulzi

Kangsheng Tu

Thomas Tu

Anastasios Tzingounis

Thomas Urup

Arjen Van Der Schaaf

Stefaan Van Gool

B Van Ness

Juliette Vergnaud

J Villanueva

Tong Hong Wang

Chaolong Wang

Qianghu Wang

Keming Wang

Chao Wang

Mei Wang

Noel Warfel

K Watabe

Massayuki Watanabe

Joshua Waterfall

Gregory Way

Yang Wei

Anton Wellstein

Eva Maria Wenzel

Adrian P Wiegmans

John Wiencke

Margareta Wilhelm

I Wolf

Vincent Kam Wai Wong

Kwan Yeung Wong

Wendy Woodward

Yadi Wu

Kai Liang Wu

FT Wunderlich

Limin Xia

Zhi‐Xiang Xu

Liyan Xu

Tao Xu

Wenrong Xu

Xiao‐Mei Yang

Runkuan Yang

Bowen Yao

Wantong Yao

Yosef Yarden

Dan Ye

Laising Yen

Wenjun Yi

Jianming Ying

Xiao Ying

Hirofumi Yoshino

Yasuaki Tamura

Guido JR Zaman

Marina Zemskova

Tianzuo Zhan

Shi‐Neng Zhang

Yinghui Zhang

Long Zhang

Hua Zhang

Cheng Cheng Zhang

Zhaolei Zhang

L Zhang

Xiaohui Zhao

Peiqing Zhao

Yang Zhao

Lei Zheng

Shaoquan Zheng

Hanqiu Zheng

Haoming Zhou

Qingyu Zhou

Guangqian Zhou

Naiqiang Zhu

Hua Zhu

Xiaodong Zhu

Xun Zhu

Wei Zhuo

Shanbeh Zienolddiny

Eugenio Zoni

